# Clinical Features, Diagnostic Test Performance, and Prognosis in Different Subtypes of Chronic Pulmonary Aspergillosis

**DOI:** 10.3389/fmed.2022.811807

**Published:** 2022-02-11

**Authors:** Huanhuan Zhong, Yaru Wang, Yu Gu, Yueyan Ni, Yu Wang, Kunlu Shen, Yi Shi, Xin Su

**Affiliations:** ^1^Department of Respiratory and Critical Care Medicine, Jinling Hospital, Medical School of Nanjing University, Nanjing, China; ^2^Department of Respiratory and Critical Care Medicine, Jinling Hospital, Nanjing Medical University, Nanjing, China; ^3^Department of Respiratory and Critical Care Medicine, Jinling Hospital, Southern Medical University, Guangzhou, China

**Keywords:** chronic pulmonary aspergillosis, clinical features, prognosis factors, surgery, antifungal duration

## Abstract

**Objective:**

The aim of this study was to describe clinical features in different subtypes of chronic pulmonary aspergillosis (CPA)-simple aspergilloma (SA), chronic cavitary pulmonary aspergillosis (CCPA), chronic fibrosing pulmonary aspergillosis (CFPA), aspergillus nodule (AN), and subacute invasive aspergillosis (SAIA), respectively, and identify long-term prognosis of CPA.

**Methods:**

We reviewed patients diagnosed with different subtypes of CPA from 2002 to 2020 at Nanjing Jinling Hospital, China. We analyzed the clinical and survival information of five different subgroups. A Cox regression model was used to explore proper antifungal duration and long-term survival factors of CCPA and SAIA.

**Results:**

A total of 147 patients with CPA were included, consisting of 11 SA, 48 CCPA, 5 CFPA, 12 AN, and 71 SAIA. The most common underlying pulmonary disease was pulmonary tuberculosis (*n* = 49, 33%), followed by bronchiectasis (*n* = 46, 31.3%) and chronic obstructive pulmonary disease (COPD) or emphysema (*n* = 45, 30.6%), while in SAIA and CFPA groups, the most common was COPD or emphysema (45.1 and 100%). Cough (85%), expectoration (70.7%), hemoptysis (54.4%), and fever (29.9%) were common symptoms, especially in CCPA, CFPA, and SAIA groups. The common imaging manifestations included cavitation (*n* = 94, 63.9%), fungal ball (*n* = 54, 36.7%), pleural thickening (*n* = 47, 32.0%), and bronchiectasis (*n* = 46, 31.3%). SAIA and CFPA groups had a lower value of hemoglobin (HB) and serum albumin (ALB) with higher C-reactive protein and erythrocyte sedimentation rate. The positive rate of sputum culture, serum galactomannan (GM), and bronchoalveolar lavage fluid GM was 32.7% (36/110), 18.4% (18/98), and 48.7% (19/39), respectively. There were 64.6% (31/48) patients with CCPA and 25.4% (18/71) patients with SAIA who received surgery and the 5-year cumulative survival rate was 92.1 and 66.6%, respectively. SAIA, old age, male, low body mass index (BMI), COPD or emphysema, multiple distribution, low serum ALB, and positive sputum culture were adverse prognosis factors for SAIA and CCPA group, and BMI ≤ 20.0 kg/m^2^ was independently associated with increased mortality (hazard ratio (*HR*) 5.311, 95% *CI* 1.405–20.068, *p* = 0.014). Multivariable Cox regression indicated that surgery (*HR* 0.093, 95% *CI* 0.011–0.814, *p* = 0.032) and antifungal duration >6 months (*HR* 0.204, 95% *CI* 0.060–0.696 *p* = 0.011) were related to improved survival.

**Conclusion:**

The clinical features and laboratory test performance are different among SA, CCPA, CFPA, AN, and SAIA. Low BMI was an independent risk factor for survival. Selective surgery and antifungal duration over 6 months were associated with improved survival.

## Introduction

Chronic pulmonary aspergillosis (CPA) was thought to affect over 3 million people worldwide (1). It is caused by the Aspergillus species, usually, *Aspergillus fumigatus*, whose conidia are present in indoor and outdoor environments and tiny enough (2–3 μm) to be inhaled into the small airway (2). CPA always complicates many other respiratory disorders such as pulmonary tuberculosis, non-tuberculous mycobacterial infection, allergic bronchopulmonary aspergillosis (ABPA), and chronic obstructive pulmonary disease (COPD) or emphysema (3, 4). If not been treated properly, it may progressively exacerbate.

According to the European guidelines by Denning et al. in 2016 (4), CPA is divided into 5 different subtypes. Chronic cavitary pulmonary aspergillosis (CCPA) is one of the most common, if untreated, which may progress to chronic fibrosing pulmonary aspergillosis (CFPA). Simple aspergilloma (SA) and Aspergillus nodule (AN) are less common and have few symptoms. The above four categories are often found in non-immunocompromised patients, while subacute invasive aspergillosis (SAIA), a more rapidly progressive infection, is usually found in moderately immunocompromised individuals. Since Denning et al. proposed the above guideline, only few small simple studies described all ungrouped CPA cases in China (5). However, the definition of CPA, especially with regard to the classification of subtypes, still remains uncertain, and the therapy and outcome of these subtypes are different. Therefore, it is necessary for us to further identify the characteristics and prognosis of different subtypes to guide proper clinical treatment, especially between CCPA and SAIA which are similar in symptom and imaging. Recently, there are quite a few studies trying to clarify the risk factors affecting the prognosis of patients with CPA, and a 5-year survival rate range from 45 to 62% (6–8), but no exact consensus is come up with. Furthermore, the optimal antifungal duration is still unclear so far. The aim of this study was to describe clinical features and diagnostic performance in different subtypes of CPA, identify potential prognostic factors for 10-year survival and explore proper anti-fungal duration especially in CCPA and SAIA groups.

## Materials and Methods

### Study Design

This retrospective study reviewed hospitalized patients with CPA referred to the Nanjing JinLing Hospital from January 2002 to December 2020. In addition, patients were reassessed by at least two professors of respiratory medicine. The survival data were finally collected in June 2021 or follow-up time reached 10 years, resulting in a minimum survival data of 6 months for surviving patients. The diagnosis and classification of CPA were based on the European guidelines published in 2016 by Denning et al. All patients had the following characteristics (4): (1) one or more cavities with or without a fungal ball present or nodules on thoracic imaging; (2) microbiological evidence of Aspergillus infection (microscopy or culture from biopsy, sputum, or bronchoalveolar lavage fluid (BALF) culture, positive serum or BALF galactomannan (GM) test or serum anti-Aspergillus immunoglobulin G (IgG) antibody and exclusion of alternative diagnoses; and (3) all present for at least 3 months (SAIA may present for 1–3 months). Patients who did not fulfill these diagnostic criteria were excluded.

According to clinical and radiological findings, patients were divided into 5 different subtypes including SA, CCPA, CFPA, AN, and SAIA. The specific classification methods were as follows: (1) SA occurred in a non-immunocompromised group and presented as a single pulmonary cavity or bronchiectasis containing a fungal ball on imaging. They had mild symptoms such as cough, sputum, and hemoptysis or no symptom and had no radiological progression over at least 3 months of observation. (2) Patients with CCPA presented as one or more pulmonary cavities containing one or more aspergillomas or irregular intraluminal material and had radiological progression such as new cavities, increasing pericavitary infiltrates, or increasing fibrosis. They often had significant pulmonary and/or systemic symptoms such as cough, sputum, hemoptysis, or fever and lasted for at least 3 months, but the disease progression was slower than SAIA. (3) CFPA suffered severe fibrotic destruction of at least two lobes of the lung which manifested as consolidation, reduced volume, or large cavities with surrounding fibrosis on imaging. They all had very poor lung function and significant pulmonary and systemic symptoms. Those who involved only one lobe were classified as CCPA. (4) AN presented as one or more nodules on imaging. They often had minor or no symptoms and could only be definitively diagnosed by histology in which aspergillus complicating necrosis could be seen but there was no tissue invasion. (5) SAIA had a similar clinical manifestation as invasive pulmonary aspergillosis (IPA) but the disease progression was slower than that and the natural course lasted for 1–3 months. They usually occurred in mildly immunocompromised patients, such as diabetes or use of glucocorticoids or immunosuppressants, and had variable radiological features, including cavitation, nodules, infiltrates, or consolidation. The biopsy of SAIA shows hyphae in invading lung tissue.

We performed an analysis of the baseline characteristic, clinical, and radiological manifestation, laboratory and microbiology data, treatment and survival, especially surgery and anti-fungal duration information within 10 years of patients with CPA in different subtypes. The baseline collected was before or at admission. Positive serum or BALF GM test was defined as ≥1.0 pg/ml (ELISA, Bio-Rad Laboratories, CA, USA), and positive aspergillus-specific IgG antibody was >80 AU/ml (ELISA, Dynamiker, China). Due to CCPA and SAIA groups being the most common form of CPA, and deaths of CCPA and SAIA account for almost 90% of all, it is significant to conduct the survival analysis to explore risk factors for prognosis among the above two groups. The study protocol was approved by the Institute Ethics Committee of Nanjing Jinling Hospital. Informed consent was waived because of the retrospective nature of the study.

### Statistical Analysis

Data are shown as mean ± *SD* in normal distribution or median with interquartile range (*IQR*) in non-normal distribution for quantitative variables and as numbers (percentages) for qualitative variables. The chi-square and Fisher's exact tests were used for categorical variables. Quantitative variables with normal distributions were compared with Student's *t*-test, while non-normally distributed variables were compared with the Mann–Whitney *U*-test. Survival curves were used to analyze the prognosis of patients from diagnosis to year 10. Survival analysis was performed with the Kaplan–Meier method with the log-rank test. A multivariable Cox analysis was performed to explore independent risk factors for 10-year mortality in CCPA and SAIA groups. An effect was considered to be statistically significant when the *p*-value was < 0.05, and all significance tests were two-tailed. The data were statistically analyzed using SPSS 25.0.

## Results

### Clinical Characteristics

A total of 147 patients proven with CPA were included in this study. The most common subtypes of CPA were SAIA (*n* = 71) and CCPA (*n* = 48) in this study, followed by AN (*n* = 12), SA (*n* = 11) and CFPA (*n* = 5). The baseline characteristics of CPA in different subtypes are summarized in Table 1. There were 85 men and 62 women with an average age of 55.5 (18–89) years. In CCPA, CFPA, and SAIA groups, men were in the majority, and patients with CFPA were older than other subtypes. The mean body mass index (BMI) was 21.9 ± 3.0 kg/m^2^, and the CFPA and SAIA groups had lower BMI.

**Table 1:** Baseline characteristics of patients with chronic pulmonary aspergillosis (CPA) in different subtypes.

**Characteristic**	**ALL** **(***n*** = 147)**	**SA** **(***n*** = 11)**	**CCPA** **(***n*** = 48)**	**CFPA** **(***n*** = 5)**	**AN** **(***n*** = 12)**	**SAIA** **(***n*** = 71)**
Sex, male	85 (57.8)	4 (36.4)	27 (56.3)	4 (80.0)	4 (33.3)	46 (64.8)
Age, years	55.5 ± 13.8	46.5 ± 13.7	51.8 ± 14.0	60.6 ± 6.8	51.3 ± 11.5	59.7 ± 13.0
BMI, kg/m^2^	21.9 ± 3.0	22.4 ± 1.6	22.0 ± 2.9	20.9 ± 4.9	23.2 ± 3.1	21.6 ± 3.2
Underlying pulmonary disease, *n* (%)	104 (70.7)	4 (36.4)	34 (36.4)	5 (100.0)	5 (41.7)	56 (78.9)
Tuberculosis	49 (33.3)	2 (18.2)	20 (41.7)	3 (60.0)	3 (25.0)	21 (29.6)
Bronchiectasis	46 (31.3)	2 (18.2)	9 (18.8)	4 (80)	2 (16.7)	29 (40.8)
COPD or/and emphysema	45 (30.6)	1 (9.1)	6 (12.5)	5 (100)	1 (8.3)	32 (45.1)
ABPA	3 (2.0)	0	1 (2.1)	0	0	2 (2.8)
Asthma	1 (0.7)	0	0	0	0	1 (1.4)
Lung cancer	6 (4.1)	0	0	0	1 (8.3)	5 (7.0)
History of pulmonary surgery	3 (2.0)	0	2 (4.2)	0	0	1 (1.4)
Others	5 (3.4)	0	2 (4.2)	0	0	3 (4.2)
**Underlying systemic disease**, ***n*** **(%)**
Diabetes	17 (11.6)	0	1 (2.1)	1 (20.0)	0	15 (21.1)
Circulation system disease	24 (16.3)	1 (9.1)	3 (6.3)	1 (20.0)	3 (25.0)	16 (22.5)
Chronic hepatitis	7 (4.8)	0	2 (4.2)	1 (20.0)	0	4 (5.6)
Extra-pulmonary malignancy	10 (6.8)	1 (9.1)	2 (4.2)	0	0	7 (9.9)
Autoimmune disease	14 (9.5)	0	5 (10.4)	1 (20.0)	1 (8.3)	7 (9.9)
Ankylosing spondylitis	5 (3.4)	0	3 (6.3)	0	0	2 (2.8)
Long term use of glucocorticoid or immunosuppressants (over 3weeks)	13 (8.8)	0	3 (6.3)	0	1 (8.3)	9 (12.7)

*BMI, body mass index; COPD, chronic obstructive pulmonary disease; ABPA, allergic bronchopulmonary aspergillosis*.

Approximately, 82.0% (121) of patients had either underlying pulmonary disease or systematic disease, the most common was pulmonary tuberculosis (*n* = 49, 33.0%) including 5 patients with existing tuberculosis and 45 cases with previous tuberculosis, followed by bronchiectasis (*n* = 46, 31.3%) and COPD or emphysema (*n* = 45, 30.6%). Tuberculosis was the most common pulmonary disease in CCPA group (*n* = 20, 41.7%), so it was in SA (*n* = 2, 18.2%) and AN (*n* = 3, 25.0%), while in SAIA group was COPD or emphysema (*n* = 32, 45.1%) which all 5 patients with CFPA also suffered. Six patients with lung cancer were found in CPA, one of them had coexistent AN, and five were diagnosed with SAIA after chemotherapy or radiotherapy. A high rate of diabetes was also found (*n* = 17, 11.6%) mainly in SAIA group (*n* = 15, 21.1%). Fourteen patients suffered autoimmune disease, such as ankylosing spondylitis, and 13 patients had received long-term (>3 weeks) glucocorticoid or immunosuppressants therapy.

The Clinical and Radiological manifestation data were shown in Table 2. The most common symptoms were cough (*n* = 125, 85%) and expectoration (*n* = 104, 70.7%), followed by hemoptysis (*n* = 80, 54.4%) and fever (*n* = 44, 29.9%), most of these symptoms focused on CCPA, CFPA, and SAIA groups, while occasionally occurring in the SA and AN groups. Hemoptysis was more common in the CCPA group than that in SAIA (70.4 vs. 52.1%, *p* < 0.05), while fever was mainly seen in the SAIA group compared with CCPA (43.7 vs. 27.1%, *p* = 0.066). Cavitation (*n* = 94, 63.9%) was the most common imaging finding in CPA, followed by fungal ball (*n* = 54, 36.7%), pleural thickening (*n* = 47, 32.0%), and bronchiectasis (*n* = 46, 31.3%). Sixty-five patients (44.2%) presented with a single lesion on imaging, while eighty-two (55.8%) with multiple lesions. Except in SA and AN groups, multiple distribution is more common in other groups.

**Table 2:** The clinical and radiological manifestation of patients with CPA in different subtypes.

**Clinical feature**	**ALL**	**SA**	**CCPA**	**CFPA**	**AN**	**SAIA**	***P*** **-value**
	***n*** **= 147**	***n*** **= 11**	***n*** **= 48**	***n*** **= 5**	***n*** **= 12**	***n*** **= 71**	
**Clinical manifestations**, ***n*** **(%)**
Cough	125 (85.0)	4 (36.4)	44 (91.7)	5 (100.0)	5 (41.7)	67 (94.4)	
Sputum	104 (70.7)	2 (18.2)	35 (72.9)	5 (100)	3 (25.0)	59 (83.1)	
Hemoptysis	80 (54.4)	1 (9.1)	34 (70.8)	3 (60.0)	5 (41.7)	37 (52.1)	0.041^#^
Fever	44 (29.9)	0	13 (27.1)	0	0	31 (43.7)	0.066^#^
Chest distress/asthma/dyspnea	46 (31.3)	0	7 (14.6)	4 (80.0)	2 (16.7)	33 (46.5)	
Chest pain	22 (15.0)	1 (9.1)	7 (14.6)	1 (20.0)	1 (8.3)	12 (16.9)	
Weak or night sweat	14 (9.5)	0	6 (12.5)	1 (20)	2 (16.7)	5 (7.0)	
**Imaging manifestations**, ***n*** **(%)**
Cavitation	94 (63.9)	7 (63.6)	48 (100.0)	4 (80.0)	1 (8.3)	34 (47.9)	
Fungal ball	54 (36.7)	11 (100.0)	23 (47.9)	4 (80.0)	3 (25.0)	13 (18.3)	
Nodule	31 (21.1)	0	9 (18.8)	1 (20.0)	12 (100.0)	9 (12.7)	
Consolidation	16 (10.9)	0	4 (8.3)	1 (20.0)	0	11 (15.5)	
Pleural thickening	47 (32.0)	1 (9.1)	20 (41.7)	3 (60.0)	2 (16.7)	21 (29.6)	
Bronchiectasis	46 (31.3)	2 (18.2)	9 (18.8)	4 (80)	2 (16.7)	29 (40.8)	
Single lesion	65 (44.2)	11 (100.0)	22 (45.8)	0	9 (75.0)	23 (32.4)	
Multiple lesion	82 (55.8)	0	26 (54.2)	5 (100.0)	3 (25.0)	48 (67.6)	

# *Means comparing between CCPA and SAIA*.

Laboratory and microbiology data were summarized in Table 3. Inflammation indicators including C-reactive protein (CRP, *n* = 115) and erythrocyte sedimentation rate (ESR, *n* = 65) significantly increased in the SAIA group compared with CCPA, SA, and AN groups (CRP *p* < 0.001, ESR *p* = 0.017), and the CFPA group also had a high level above normal. The patients with CFPA (105 g/L, *IQR* 99–138) and SAIA (117 g/L, *IQR* 105–126) both had lower hemoglobin (HB), while the other three groups had significantly higher HB. The median albumin (ALB) was 33.5 (*IQR* 32.4–41.8) g/L and 36.5 (*IQR* 28.0–47.1) in CFPA and SAIA groups, respectively, which was lower than that in SA, CCPA, and AN groups (SAIA *p* < 0.001, CFPA *p* = 0.036).

**Table 3:** Laboratory and microbiology data of patients with CPA in different subtypes.

**Variable**	**ALL** **(***n*** = 147)**	**SA** **(***n*** = 11)**	**CCPA** **(***n*** = 48)**	**CFPA** **(***n*** = 5)**	**AN** **(***n*** = 12)**	**SAIA** **(***n*** = 71)**	***P*** **-value**
**Laboratory data, median (IQR)**							
WBC count, 10^9^/L (146)	6.2 (4.9–8.0)	5.5 (4.4–6.2)	5.8 (4.5–7.2)	7.8 (6.7–9.8)	5.3 (4.6–6.0)	6.9 (5.2–8.8)	
Neutrophil count	3.6 (2.8–5.8)	3.3 (2.0–4.0)	3.1 (2.6–5.2)	5.5 (4.4–6.9)	3.1 (2.2–3.6)	4.1 (3.0–6.9)	
HB, g/L (146)	123 (111–136)	131 (115–139)	131 (119–139)	105 (99–138)	133 (119–144)	117 (105–126)	
ALB, g/L (147)	40.5 (33.5–44.3)	42.7 (40.7–44.0)	42.2 (37.9–45.2)	33.5 (32.4–41.8)	42.5 (40.0–46.9)	36.5 (28.0–47.1)	<0.001
CRP, mg/L (115)	4.1 (1.0–28.2)	1.0 (0.5–2.5)	2.6 (0.6–17.0)	19.1 (8.7–57.6)	0.9 (0.6–2.7)	13.8 (2.5–80.6)	<0.001
ESR, mm (65)	34 (16.5–70.5)	4 (3–4)	22 (12.5–57.0)	47 (/)	/	39 (27–80)	0.017
**Microbiology data**, ***n*** **(%)**							
Sputum culture (110)	36/110 (32.7)	0/3	11/34 (32.4)	1/4 (25.0)	2/7 (28.6)	22/62 (35.5)	
Serum GM (102)	18/98 (18.4)	1/5 (20.0)	7/33 (21.2)	1/4 (25.0)	0/6	9/50 (18)	
BALF GM (39)	19/39 (48.7)	0/2	9/14 (64.3)	0/1	1/3 (33.3)	9/19 (47.4)	
1,3-β-D-glucan (BDG) (91)	30/91 (33.0)	0/4	8/22 (36.4)	2/5 (40.0)	0/5	20/55 (36.4)	
NGS or PCR (7)	7	1	1	0	0	5	
Pathology (86)	86	10	36	0	12	28	
Aspiration biopsy	27	1	8	0	5	13	
Surgical excision	66	9	31	0	8	18	
Aspergillus specific IgG antibody, AU/ml (24)	125.33 (47.97–293.96)	/	70.57 (33.88–163.35)	/	/	312.76 (270.03–757.57)	0.010^#^
Aspergillus specific IgG antibody, *n*	13/24	0/1	4/11	1/1	1/3	7/8	

*IQR, interquartile range; WBC, white blood cell; HB, hemoglobin; ALB, albumin; ESR, erythrocyte sedimentation rate; CRP, C-reactive protein; GM, galactomannan; G BALF, bronchoalveolar lavage fluid; NGS, next generation sequencing; PCR, polymerase chain reaction*.

# *Means comparing between CCPA and SAIA*.

Pathology evidence was found among 86 patients by lung biopsy (*n* = 27) and/or surgical excision (*n* = 66). Sputum culture was performed in 110 patients and was found positive in 36 (32.7%) cases, including 22 (35.5%) patients with SAIA and 11 (32.4%) patients with CCPA. Approximately, 98 patients performed serum GM test and had 18 (18.4%) positive results. BALF GM test was performed in 39 patients mainly with CFPA and SAIA, the positive rate was 48.7% which was higher than serum GM (*p* < 0.001) and sputum culture (*p* = 0.075), and the CCPA group had a similar positive rate of BALF GM with SAIA (64.3 vs. 47.4%, *p* = 0.335). Unfortunately, there were only 24 patients who conducted Aspergillus-specific IgG antibody test and the median was 125.33 (*IQR* 47.97–293.96) AU/ml, and unexpectedly, the level in patients with SAIA (312.75 AU/ml, *IQR* 270.03–757.57) was significantly higher than patients with CCPA (70.57 AU/ml, *IQR* 33.88–163.35) (*p* = 0.010).

### Therapy and Prognostic Factors

As was shown in Table 4, antifungal treatment was administrated in 83% (122 out of 147 patients) patients with CPA, including 24 cases before surgery, 20 after surgery, and 78 cases using the drug alone. The most two common first-line anti-fungal drugs were voriconazole (*n* = 70, 57.4%) and itraconazole (*n* = 35, 28.7%), 7 patients changed triazoles from itraconazole to voriconazole during treatment. The duration of 32 cases (30.5%) lasted over 6 months, while 73 cases (69.5%) were <6 months. Surgery was performed in 66 patients (44.9%). The proportion of surgery is high especially in the patients with SA (81.8%), CCPA (64.6%), and AN (66.7%).

**Table 4:** Treatment and outcomes of patients with CPA in different subtypes.

**Variable**	**ALL** **(***n*** = 147)**	**SA** **(***n*** = 11)**	**CCPA** **(***n*** = 48)**	**CFPA** **(***n*** = 5)**	**AN** **(***n*** = 12)**	**SAIA** **(***n*** = 71)**
**Treatment method**						
Drug alone	78	2	17	4	3	52
Surgery alone	16	5	8	0	2	1
Drug + surgery	44	4	21	0	5	14
Before surgery	24	1	13	0	3	7
After surgery	20	3	8	0	2	7
Surgery therapy	66	9	31	0	8	18
Lobectomy	50	6	26	0	5	13
Wedge resection	9	3	3	0	2	1
Multiple lobectomy	2	1	0	0	0	1
Unilateral pneumonectomy	1	0	0	0	1	0
Unknown	4	0	1	0	0	3
Antifungal drug						
VCZ	70	4	23	4	5	33
ITZ	35	0	11	0	3	21
Changing triazoles	7	0	1	0	0	6
Others or unknow	10	2	2	0	0	7
Never antifungal treatment	18	5	8	1	3	1
Antifungal time, months						
≤6	73	5	23	2	6	37
>6	32	0	8	2	2	20
Deaths	33	1	3	3	1	25

*VCZ, voriconazole; ITZ, itraconazole*.

Among all 147 patients, we successfully followed up 140 patients from diagnosis to 10-year or June 2021. While 7 patients lost follow-up completely. During the follow-up, 34 patients died, including 26 cases of SAIA, 3 of CCPA, 3 of CFPA, one of SA, and one of AN, respectively. In the AN group, only one patient died because of unilateral pneumonectomy which was excessive treatment for this case. For patients with SA followed up, there was only one case complicating tuberculosis dead because of massive hemoptysis. However, 60% of patients with CFPA died by the end of follow-up time.

**Figure 1:**
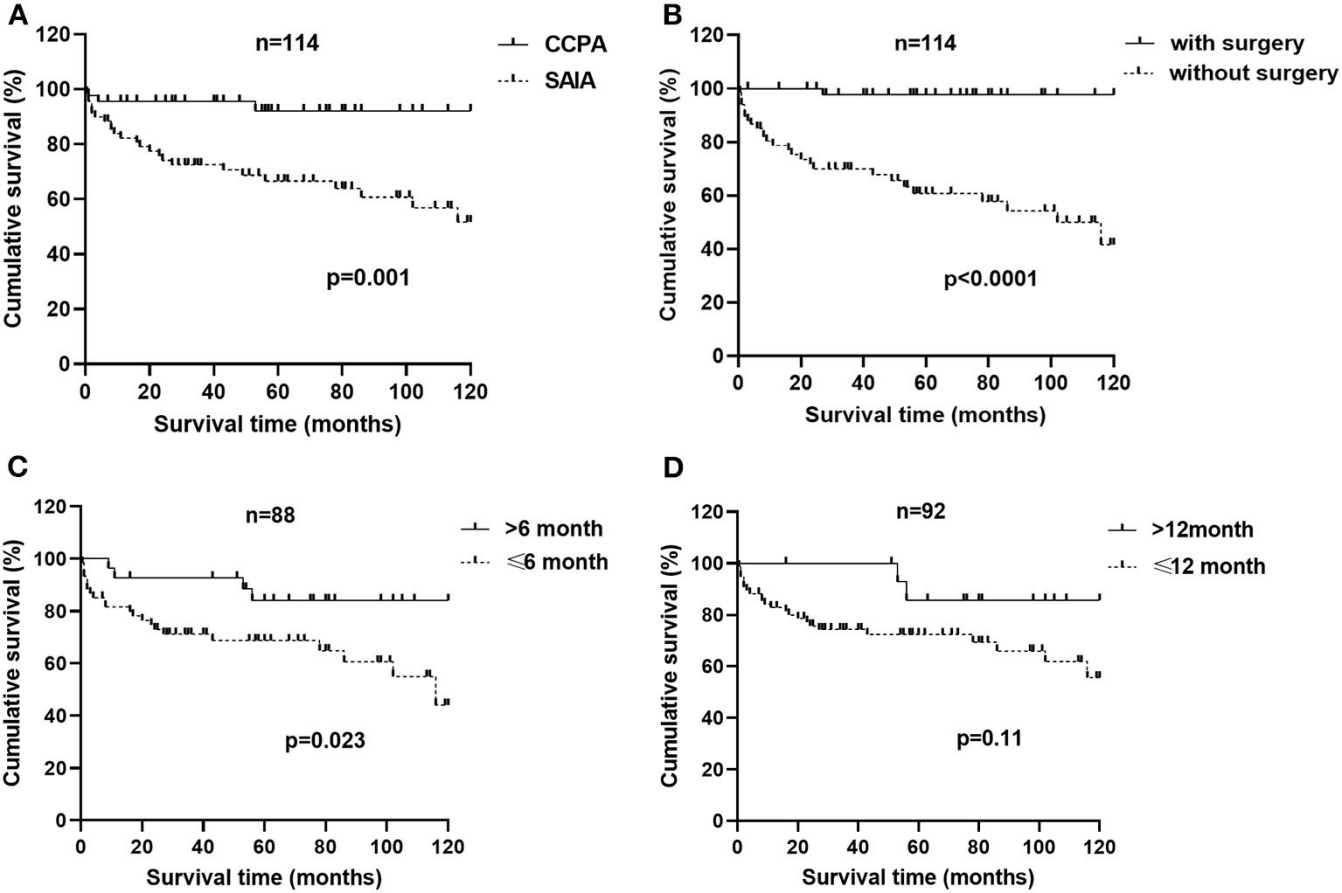
Factors associated with a 10-year survival of patients with chronic cavitary pulmonary aspergillosis (CCPA) and subacute invasive aspergillosis (SAIA). **(A)** Survival from diagnosis to month 120 in patients of CCPA and SAIA groups according to Kaplan–Meier (K–M) analysis with log-rank test (*p* = 0.001). **(B)** K–M cumulative survival curve of patients with chronic pulmonary aspergillosis (CPA) with and without surgical therapy with the log-rank test (*p* < 0.0001). **(C)** K–M cumulative survival curve of patients with CPA who accept anti-fungal therapy for ≤6 months and >6months with log-rank test (*p* = 0.023). **(D)** K–M cumulative survival curve of patients with CPA who accept anti-fungal therapy for ≤ 12 months and >12months with log-rank test (*p* = 0.11).

**Figure 2:**
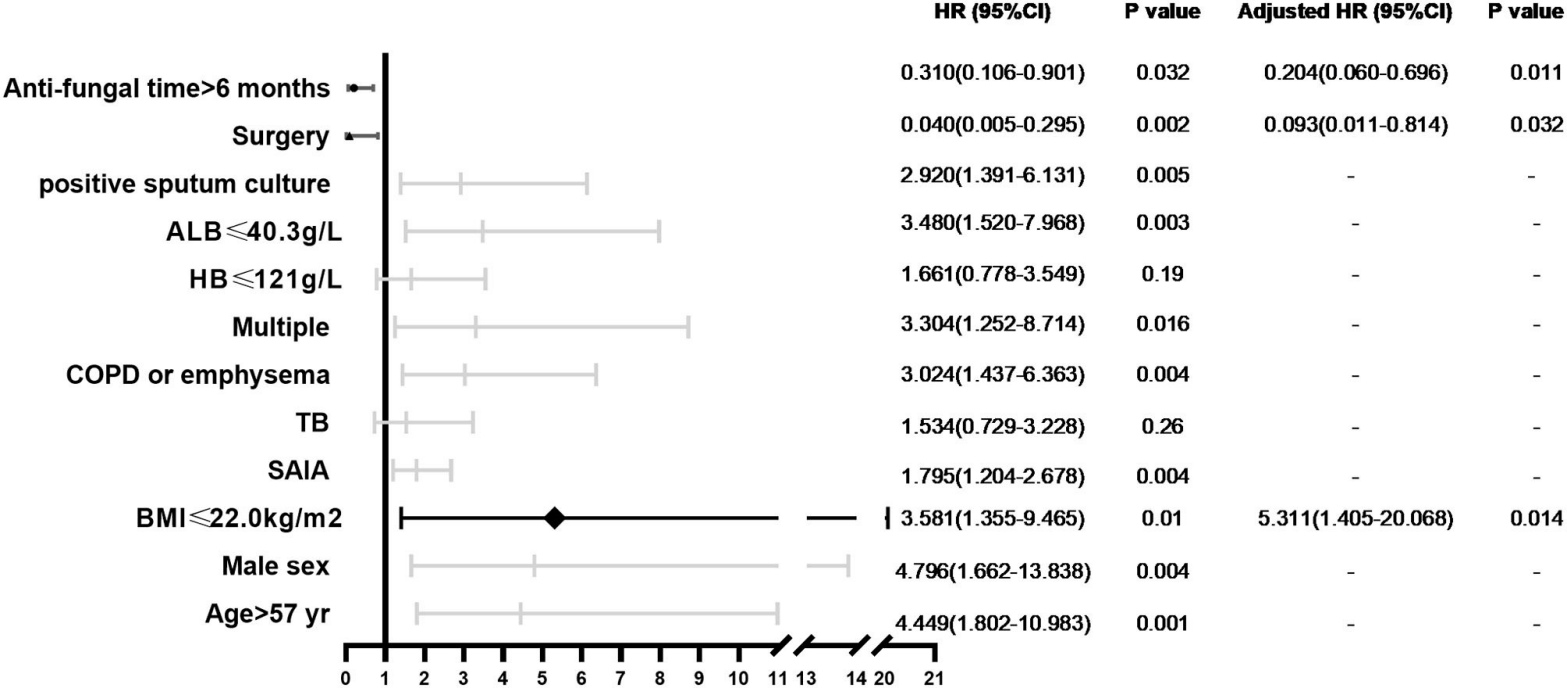
Forest plot of prognostic factors independently associated with 10-year mortality in patients with CCPA and SAIA.

We analyzed 10-year survival-related factors in the CCPA and SAIA groups because they were the most common subtypes of CPA in this study. The Kaplan–Meier cumulative survival curve of different factors in CCPA and SAIA groups was presented in Figure 1. As we can see, the cumulative survival rate was significantly lower in the SAIA group than in the CCPA group (log-rank test, *p* = 0.001, Figure 1A), and the 1-, 5-, and 10-year cumulative survival rates in the CCPA group were 95.6, 92.1, and 92.1%, respectively, and 82.3, 66.6, and 51.8%, respectively, in SAIA group. According to the Univariate Cox regression shown in Figure 2, age > 57 years, male, BMI ≤ 22.0 kg/m^2^, SAIA, COPD or emphysema, multiple distributions on imaging, ALB ≤ 40.3 g/L, and positive sputum culture were the risk factors for 10-year mortality.

In CCPA and SAIA group, 45 patients accepted surgical treatment, 60 cases were treated by antifungal drugs for <6 months and 28 cases beyond 6 months and Cox regression indicated that surgery and antifungal duration > 6 months were protective factors. As was presented in Figure 1, the cumulative survival rates of patients treated with surgery and/or antifungal duration > 6 months were significantly higher than those without surgery or antifungal duration ≤6 months groups (log-rank test, *p* < 0.0001 and *p* = 0.023, Figures 1B,C). Although antifungal duration > 12 months had lower mortality than duration ≤ 12 months (Figure 1D), we found no statistical difference finally (*p* = 0.11). There was no significant difference in overall mortality between voriconazole and itraconazole antifungal therapy. Further adjusting confounding factors with multivariable Cox regression, we found that BMI ≤ 20.0 kg/m^2^ (*HR* 5.311, 95% *CI* 1.405–20.068, *p* = 0.014) was still independently associated with higher 10-year mortality, meanwhile, surgery (*HR* 0.093, 95% *CI* 0.011–0.814, *p* = 0.032) and antifungal duration >6 months (*HR* 0.204, 95% *CI* 0.060–0.696 *p* = 0.011) were related to improved survival.

## Discussion

Chronic pulmonary aspergillosis is a potential chronic pulmonary infection disease, which includes SA, CCPA, CFPA, AN, and SAIA, and the overlap of them is often seen (4). In this retrospective study involving 147 patients, we illustrated the characteristics of five subtypes and explored the risk factors for the long-term prognosis of patients with CCPA and SAIA.

The distribution of the CPA subgroup varied in different areas. Many research studies indicated that CCPA was the most common type of CPA (4, 9, 10), but in this study, the most common was SAIA (48.3%), and CCPA accounted for 32.7%, followed by SN (8.2%), SA (7.5%), and CFPA (3.4%). Previous tuberculosis (33.3%) was the most common underlying disease in CPA, especially in the CCPA group, which was similar to some previous studies (3, 4, 6, 9, 11). In recent years, COPD and emphysema became increasingly common in patients with CPA (7, 8, 12), we also found that COPD or emphysema was the highest-incidence disease in SAIA and CFPA groups (45.1 and 100%, respectively). As we know, SAIA often occurred in the mild-immunosuppression population (4), a moderate rate of diabetes (21.1%), and autoimmune disease (9.9%) were also found in the SAIA group. It is noticeable that subtle immunodeficiency seems to be present in some patients with CCPA too. Colombo et al. found that patients with CPA show impaired IFN-γ production in peripheral blood in response to stimuli, and immunotherapy with IFN-γ could be beneficial for those patients (13), which was consistent with what Kelleher et al. shared before (14). We should pay attention to this in the future. Non-tuberculous mycobacteria (NTM) pulmonary disease was common in CPA (15), but our study was lack of information about it, which may result in ignoring patients combined with NTM infection indeed.

Clinical presentation of CPA is non-specific, and hemoptysis is the most common and life-threatening symptom (4, 16, 17). However, we found that the incidence of hemoptysis ranked third in our study (54.4%), and the most common symptom was cough (85.0%). Among all five groups, hemoptysis was more often seen in the CCPA group (70.8%) while fever was more frequently seen in SAIA (43.7%). In SA and SN groups, cough and hemoptysis were also common, but 11 out of 23 patients (47.8%) had no clinical symptom, whose lesions were found by healthy examination occasionally and were diagnosed by surgical resection. Radiological presentation of CPA based on cavitation with or without aspergilloma (4). In our study, common imaging findings of CPA were cavitation, fungal ball, pleural thickening, and bronchiectasis. SA usually had only one cavity with aspergilloma on imaging which could be distinguished from other groups easily. CFPA involved multiple lung lobes and manifested as fibrosis or consolidation, which was consistent with our study. SN presented as a single (9 out of 12 patients, 75%) or multiple nodules (3 out of 12 patients, 25%), but it is difficult to distinguish them from other lung pathology on CT findings alone and we can only rely on pathology finally (4, 18). Sometimes, SAIA and CCPA are difficult to distinguish from each other based on a single radiology result. CCPA may present as pre-existing cavities showing peri-cavitary infiltrates and SAIA commonly had progressive lesions within weeks (19).

Inflammation factors such as WBC count, CRP, and ESR are not always high in CPA. Usually, they increased in CCPA, CFPA, and SAIA groups along with systemic symptoms, while that in patients with SA and AN had no increase (4). We found that SAIA and CFPA groups had more pronounced abnormalities in blood test results such as higher CRP and ESR, lower ALB and HB; meanwhile, they were also accompanied by old age and low BMI. These findings may help us distinguish CCPA and SAIA to some degree. It is worth mentioning that Asians with CPA especially Chinese and Korean showed lower age and BMI than Europeans (5, 7, 8, 10, 12, 20–22), and this was in accordance with our study. In 2014, the American Thoracic Society highlighted the increased susceptibility to lung diseases in the older population (23). We also found CPA frequently occurred in elderly individuals and survival analysis indicated that old age (>57 years) had lower 10-year mortality in CCPA and SAIA groups. Low BMI and serum ALB usually reflect poor nutritional status. The previous studies indicated that lower BMI was a useful indicator of poor long-term survival in chronic progressive respiratory illnesses such as COPD and tuberculosis (24–26) and there were similar results for prognosis in patients with CPA (6, 7, 11, 21). Our study found that low BMI (≤20.0 kg/m^2^) is independently associated with higher 10-year mortality. Lower serum ALB was also related to a poorer outcome in patients with CCPA and SAIA in our study, which was in agreement with the previous studies (6, 7). Vanstraelen et al. illuminated that hypoalbuminemia increased unbound voriconazole plasma concentrations and possibly caused adverse events (27). Therefore, it is necessary for patients with CPA to improve nutritional status to acquire better survival, especially for older and with lower BMI CCPA patients, we should treat them more aggressively, or they may turn into CFPA eventually.

Microbiological evidence of Aspergillus infection is essential for the diagnosis of CPA. A biopsy is reliable to prove a diagnosis of pulmonary aspergillosis and was taken in 86 cases in our study. About 91% (10 out of 11) of patients with SA and all AN had biopsy evidence of microbiology. Serum GM and BALF GM tests have been demonstrated to be valuable for the diagnosis of IPA (28). For patients with CPA, the diagnosis value of the BALF GM test was better than the serum GM test (29–31), and this was also verified in our study, the positive rate of the BALF GM test (48.7%) was higher than that of serum GM test (18.4%). Sputum culture, a frequently used and traditional measure, was performed in 110 patients and the positive rate was 32.7%. All three methods mentioned above had no statistical difference between CCPA and SAIA groups. Aspergillus-specific IgG antibody test was crucial for the diagnosis of CPA and could be followed up to monitor the effectiveness of therapy (4). In our study, patients with SAIA (312.75 AU/ml) had higher antibody levels than CCPA (70.57 AU/ml), which is different from the previous studies (4, 32) probably because the sample size was too small and some antibody tests were conducted after antifungal treatment. In addition, the sensitivity of the Dynamiker Aspergillus antibody test ranged from 77 to 84.1% according to the previous studies (32–35). In recent years, new diagnostic methods such as next generation sequencing and PCR are playing an increasingly important role, but they were not routinely carried out in this study.

Antifungal drugs and surgery are two main therapies for CPA. In SA and AN groups, what we care about is whether patients need surgery or antifungal therapy. Partial previous studies recommend early surgical resection to prevent progression especially massive hemoptysis (4, 36, 37), but there was little evidence that whether patients need antifungal therapy before or after surgery, so we need more prospective study in the future. The patients with CFPA had a poor outcome, and what we need to do is control risk factors and actively treat CCPA before they deteriorate into CFPA. Voriconazole and itraconazole are the most common drugs for CPA in our study, and it is necessary to monitor therapeutic drug levels and adverse effects (4, 38).

To explore risk factors for the long-term prognosis of CCPA and SAIA and optimal antifungal treatment time, we conducted a 10-year survival analysis. The 1-, 5-, and 10-year cumulative survival rates in the CCPA group were 95.6, 92.1, and 92.1%, respectively, and 82.3, 66.6, and 51.8%, respectively, in the SAIA group, which were much higher than previous studies (6–8). The high survival rate of the CCPA group may be related to lower average age, appropriate surgical treatment, and long normative antifungal treatment in these patients. According to univariate analysis, old age, man, low BMI, COPD or emphysema, multiple distributions on imaging, low serum ALB, and positive sputum culture were all adverse prognosis factors for CPA, and patients with SAIA had a better outcome than CCPA. We found that surgery was an independent protective factor for CPA, patients with surgery had a significantly better prognosis than patients without surgery, which was consistent with the previous studies (36, 37, 39, 40). A retrospective study involving 61 patients with CPA indicated that surgical treatment accompanied with antifungal therapy before surgery could minimize the recurrence rate of CPA (41), but we did not find a significant survival difference between preoperative and postoperative antifungal therapy. In addition, some patients could not tolerate surgery due to poor cardiopulmonary function or comorbidities. Although surgery might bring a better long-term prognosis in some patients with CPA, it is necessary to carefully assess the condition of patients with CPA before making the decision. So far, there were no acknowledged criteria for treatment discontinuation in CPA, the efficacy and the adverse effects of drugs are difficult to balance and the duration of antifungal therapy remains unclear. The European CPA rationale in 2016 recommended a minimum of 4–6 months of oral triazole therapy (4). A recent retrospective study involving 196 patients from South Korea indicated that prolonging antifungal therapy beyond 12 months could reduce the recurrence rate in CPA patients (42). Another retrospective cohort from the UK of 206 patients with CPA showed that patients with extended antifungal treatment for 12 months experienced a greater improvement in the quality of life (12). In our study, we found that patients with CCPA and SAIA whose antifungal duration was beyond 6 months had a higher long-term survival rate than patients who received antifungal drugs no more than 6 months. However, we found no statistical survival difference between antifungal duration over 12 months and no more than 12 months. In general, we consider that antifungal therapy beyond 6 months was favorable for patients with CCPA and SAIA.

Our research described the clinical characteristics of CPA in different subgroups, suggested appropriate antifungal duration, and presented key prognostic factors that would assist in identifying patients at risk of poor prognosis, which could improve clinical diagnosis and treatment to some degree. But there were indeed some limitations in our study. First, this was a retrospective study conducted in a single referral hospital, and some patients lost to follow-up. Second, the diagnosis, classification, and treatment of cases many years ago are immature, which may influent the assessment of prognosis, but we remedy for it by reassessing all patients with CPA included by at least one professor of medicine. In addition, our study did not present a specific cause of death and overlooked the adverse effect of antifungal drugs. Finally, we still did not provide optimal antifungal treatment time and we need further study.

In conclusion, the clinical characteristics differ among SA, CCPA, CFPA, AN, and SAIA and clinicians can distinguish them by combining symptom, radiology, laboratory, and microbiology. SAIA, old age, male, low BMI, COPD or emphysema, multiple distributions on imaging, low serum ALB, and positive sputum culture were adverse prognosis factors for CCPA and SAIA, and low BMI was an independent risk factor after adjusting for confounding factors. Selective surgery and antifungal duration over 6 months could improve long-term survival.

## Data Availability Statement

The original contributions presented in the study are included in the article/supplementary material, further inquiries can be directed to the corresponding author/s.

## Ethics Statement

The studies involving human participants were reviewed and approved by the Institute Ethics Committee of Nanjing Jinling Hospital. Written informed consent for participation was not required for this study in accordance with the national legislation and the institutional requirements. Written informed consent was not obtained from the individual(s) for the publication of any potentially identifiable images or data included in this article.

## Author Contributions

XS, HZ, and YaW designed the study and drafted the manuscript. HZ, YaW, and YG collected the data of patients and analyzed the data. YN, YuW, and KS were critically involved in the data collection and the revision of the manuscript. YS revised the manuscript. All authors contributed to the article and approved the submitted version.

## Funding

This work was supported by the Project of Natural Science Foundation of China (82070011), the Key Project of Jiangsu Commission of Health (K2019004), and the 333 project of Jiangsu Province (BRA2019339).

## Conflict of Interest

The authors declare that the research was conducted in the absence of any commercial or financial relationships that could be construed as a potential conflict of interest.

## Publisher's Note

All claims expressed in this article are solely those of the authors and do not necessarily represent those of their affiliated organizations, or those of the publisher, the editors and the reviewers. Any product that may be evaluated in this article, or claim that may be made by its manufacturer, is not guaranteed or endorsed by the publisher.
